# Protein synthesis is associated with high-speed dynamics and broad-band stability of functional hubs in the brain

**DOI:** 10.1016/j.neuroimage.2017.04.062

**Published:** 2017-07-15

**Authors:** Peter J. Hellyer, Erica F. Barry, Alberto Pellizzon, Mattia Veronese, Gaia Rizzo, Matteo Tonietto, Manuel Schütze, Michael Brammer, Marco Aurélio Romano-Silva, Alessandra Bertoldo, Federico E. Turkheimer

**Affiliations:** a Centre for Neuroimaging Sciences, Institute of Psychiatry, Psychology and Neuroscience, King’s College London, London, UK; b Department of Bioengineering, Imperial College London, Royal School of Mines, Room 4.35, South Kensington Campus, SW7 2AZ, UK; c Department of Information Engineering, University of Padova, Padova, Italy; d Instituto Nacional de Ciência e Tecnologia em Medicina Molecular, Federal University of Minas Gerais, Belo Horizonte, Brazil; e Mental Health Department, Faculty of Medicine, Federal University of Minas Gerais, Belo Horizonte, Brazil

**Keywords:** Resting state, Protein synthesis, Functional connectivity, Graph theory, Dynamics

## Abstract

L-[1-^11^C]leucine PET can be used to measure *in vivo* protein synthesis in the brain. However, the relationship between regional protein synthesis and on-going neural dynamics is unclear. We use a graph theoretical approach to examine the relationship between cerebral protein synthesis (rCPS) and both static and dynamical measures of functional connectivity (measured using resting state functional MRI, R-fMRI). Our graph theoretical analysis demonstrates a significant positive relationship between protein turnover and static measures of functional connectivity. We compared these results to simple measures of metabolism in the cortex using [^18^F]FDG PET). Whilst some relationships between [^18^F]FDG binding and graph theoretical measures was present, there remained a significant relationship between protein turnover and graph theoretical measures, which were more robustly explained by L-[1-^11^C]Leucine than [^18^F]FDG PET. This relationship was stronger in dynamics at a faster temporal resolution relative to dynamics measured over a longer epoch. Using a Dynamic connectivity approach, we also demonstrate that broad-band dynamic measures of Functional Connectivity (FC), are inversely correlated with protein turnover, suggesting greater stability of FC in highly interconnected hub regions is supported by protein synthesis. Overall, we demonstrate that cerebral protein synthesis has a strong relationship independent of tissue metabolism to neural dynamics at the macroscopic scale.

## Highlights

• Spontaneous, neural dynamics are fundamental for information processing & function
• We combine L-[1-11C]leucine PET with Static and Dynamic measures of network topology
• ‘Hub’ brain regions are linked to increased protein synthesis, independent of tissue metabolism
• Stability of network hubs is inversely correlated with protein turnover
• Strong, stable hubs are supported by protein synthesis

## Introduction

To effectively interact with the external world, the brain must build rich representations of environmental inputs received from sensory systems and update these representations ensuing action plans to effectively interact with a dynamic environment (Turkheimer et al., 2015). Modern interpretation of brain function describes the brain as a broad network of functionally interacting regions (the functional connectome) which adapts in both space and time in response to both to external (environmental) and internal (cognitive) demands (Corbetta and Shulman, 2002, Fornito et al., 2012, Fox et al., 2005, Hellyer et al., 2015, Vincent et al., 2008). One powerful framework for exploring the functional connectome and how the disparate regions of the brain interact is graph theory. Such approaches have provided strong hypotheses about the structural organization of the cortex, identifying sets of regions that are critically important for enabling efficient neuronal signalling and communication (the so-called ‘hubs’ regions) (Hagmann et al., 2007, Sporns et al., 2004, Sporns and Honey, 2013, Sporns and Kotter, 2004, van den Heuvel et al., 2012, van den Heuvel and Sporns, 2013, van den Heuvel et al., 2013), and describing large scale connectivity networks which neatly mirror the functional connectivity of slow neural dynamics (Beckmann et al., 2005, De Luca et al., 2006, Fox et al., 2009). Fluorodeoxyglucose (^18^FDG) PET also reveals that these highly connected ‘hub’ regions are amongst the most metabolically active regions of the brain (Raichle, 2015, Raichle et al., 2001, Raichle and Snyder, 2007) supporting the hypothesis that these hubs are key, metabolic nodes responsible for large-scale ‘functional integration’ of information.

Complementary computational and experimental approaches have also provided broad evidence that the brain is functionally organized as a complex system possessing a critical attractor (Fagerholm et al., 2015, Haimovici et al., 2013, Kitzbichler et al., 2009, Meisel et al., 2013, Scott et al., 2014, Shew and Plenz, 2013, Tagliazucchi et al., 2012). A system at a ‘critical state’ is finely balanced in a position between robust ordered and chaotic dynamics. Such dynamics are attractive as a model for neural function, as they provide a mechanistic framework for storage and processing information in a fluid dynamic system (Shew and Plenz, 2013, Shew et al., 2009, Shew et al., 2011). Moreover, ‘critical’ dynamics in the brain may emerge not just by the interaction of activity between highly connected cortical regions, but also through the action of local plasticity. Homeostatic plasticity, alongside a range of other adaptive or plastic approaches, have been proposed as a potential tuning mechanism for maintaining ‘critical’ neural dynamics (Cowan et al., 2013a). In the brain, these plastic mechanisms may not only induce ‘critical’ dynamics, but also enhance the emergence of functional connectivity networks (Hellyer et al., 2016). At the same time, computational approaches have demonstrated that the removal of ‘peripheral’ and ‘hub’ nodes, defined using graph theoretical approaches in computational simulations of the brain, have differential effects on the overall dynamics of the brain (Vasa et al 2015). Thus, not only do computational accounts suggest that plasticity is key to forming flexible brain dynamics, but the network topology underlying those dynamics is important for selecting and maintaining a flexible set point for activity.

Whilst a range of works in human explores the relationship between de-novo metabolism, e.g. measured using [^18^F]FDG PET and measures of functional integration (described using graph theoretical means) – the relationship between functional integration and protein turnover which is undoubtedly associated with local cortical plasticity (such as the subtle re-organisations underlying a flexible dynamic state at rest proposed by computational accounts), has not yet been explored.

In this work, we use a graph theoretical approach (Rubinov and Sporns, 2010) to examine static and dynamic measures of functional integration and network topology at rest using functional connectivity (measured using fMRI) and measures of protein synthesis. The L-[1-^11^C]leucine PET method allows the quantitative determination of local rates of protein synthesis in the central nervous system in-vivo (Smith et al., 1988). This assay uses L-[1-^11^C]leucine as a tracer to measure the rate of incorporation of leucine, one of the nine essential amino acids, into protein. Leucine is very attractive for this kind of assay because its only pathway of degradation is transamination followed by carboxylation; here the 11C label is quickly transferred to labelled CO2 which is quantitatively minimal and negligibly re-incorporated as heavily diluted by the large pool of unlabelled CO2 resulting from carbohydrate metabolism. Hence brain radioactivity is mostly due to free L-[1-^11^C]leucine and labelled protein defining a sympathetic measure of “de novo” cerebral protein synthesis (*rCPS*) (Bishu et al., 2008, Schmidt et al., 2005, Smith et al., 2005). Applied to the human-brain this approach allows the quantification of regional plasticity in the cortex (Veronese et al., 2012).

We explore the possibility that large-scale dynamics of the brain are in part constrained empirically by plasticity at a broad range of temporal scales using both a multi-scale decomposition and dynamic functional connectivity approach. Furthermore, we compare and contrast these results with a simpler description of functional processing using ^18^FDG-PET as a measure of local metabolic demand. Whilst our analysis is broadly exploratory in nature, we test the hypothesis that functional network hubs are associated with high levels of protein synthesis, independent of basal levels of metabolism. For network hubs to maintain their stability and allow integration of flexible neural dynamics over time (predicted by computational approaches) likely requires dynamic reorganisation of the local neural architecture, thus we predict that highly interconnected hubs will be stable over time – associated with highly active protein turnover.

## Methods

### Positron emission tomography (PET)

#### [1-11C]Leucine

High-Resolution [1-^11^C]Leucine PET images were acquired from 9 healthy awake subjects (9 male, age 20–24). These data were kindly provided by the authors of a previously published study. Detailed acquisition parameters and inclusion criteria are described in detail in the original work (Bishu et al., 2008). In brief, all studies were performed on a High-Resolution Research Tomograph (HRRT) (CPS Innovations, Knoxville, TN, USA). The dynamic 90-min scan was initiated coincident with a 2-min intravenous infusion of 20–30 mCi of L-[1-^11^C]leucine. Images were reconstructed using motion-compensated three-dimensional ordinary Poisson ordered subset expectation maximization as 42 frames (16×15, 4×30, 4×60, 4×150, 14×300 s); voxel size was 1.21×1.21×1.23 mm. Concentrations of unlabelled and labelled leucine in plasma and total ^11^C and ^11^CO_2_ in whole blood were estimated using continuous arterial blood sampling. Voxel-level estimates of rCPS [nmol/gr/min] were determined by spectral analysis with an iterative filter (SAIF) (Veronese et al., 2010, Veronese et al., 2012). The resulting rCPS maps were then spatially normalized to the MNI-152 (2mm) co-ordinate system (Grabner et al., 2006) and a population average rCPS template image was calculated for further analysis. Finally, the rCPS PET template was segmented into 82 regions of interest (ROIs) according to the Desikan-Kilaney+Aseg atlas (Fischl, 2012, Fischl et al., 2002) projected into the MNI-152 coordinate space. An overview of this atlas is available in (Fig. S1), resulting in a single 82(n)x1 vector of average rCPS in each region across the cortex and sub-cortex.

#### [^18^F]-FDG (Fludeoxyglucose)

High resolution [^18^F]FDG PET images were collected from 11 healthy volunteers (5 female, age 30.5±7.1 years). [^18^F]FDG PET/CT brain images were acquired in a GE Discovery 690 (GE Healthcare, Milwaukee, Wisconsin, USA), at the Centro de Tecnologia em Medicina Molecular, Faculdade de Medicina da UFMG, Belo Horizonte, Brazil. Fifty minutes prior to scanning, each subject received an intravenous bolus injection of 5.18 MBq/kg of [^18^F]FDG before resting in a quiet, dark room with minimum stimuli. PET images were subsequently acquired over 10 minutes, and reconstructed in a 192x192x47 matrix using the OSEM (Ordered Subsets Expectation Maximization) algorithm, with 2 iterations and 20 subsets. CT images were used to perform attenuation correction. The resulting standardized uptake value (SUV) maps normalised by body weight and injected dose were then spatially normalized to the MNI-152 (2mm) co-ordinate system (Grabner et al., 2006) and a population average SUV template image was calculated for further analysis. Like the analysis described for the rCPS data above, this atlas was subsequently divided into the same 82 cortical and subcortical ROIs resulting in a single 82(n)x1 vector of average SUV in each region across the cortex and sub-cortex.

### Magnetic resonance imaging

#### Resting state fMRI (R-fMRI)

High temporal and spatial resolution R-fMRI data was obtained from 20 subjects (10 female, age 30.1±3.3 years) released as part of the Human Connectome Project (humanconnectome.org/). In order to explore the effect of gender and specific subject selection bias on the reported results, we randomly selected a cohort of a further 20 subjects (10 female, age: 30.2+3.46 years), as well as 20 male (age: 29.1+3.79 years) and 20 female subjects (age: 29.76+3.07 years) In brief, all HCP subjects were scanned on a customized Siemens 3T “Connectome Skyra” housed at Washington University in St. Louis, using a standard 32-channel Siemens receive head coil and a “body” transmission coil designed by Siemens specifically for the smaller space available using the special gradients of the WU-Minn and MGH-UCLA Connectome scanners. R-fMRI data were acquired using a Gradient-echo EPI sequence, TR=720 ms, TE=33.1 ms, flip angle 52°, multiband factor 8, slice thickness 2.0 mm for 72 slices (2.0 mm isotropic voxels) over 15 minutes – resulting in 1200 volumes. Subjects had eyes-open with fixation on a bright cross-hair on a dark background.

**Fig. 1:**
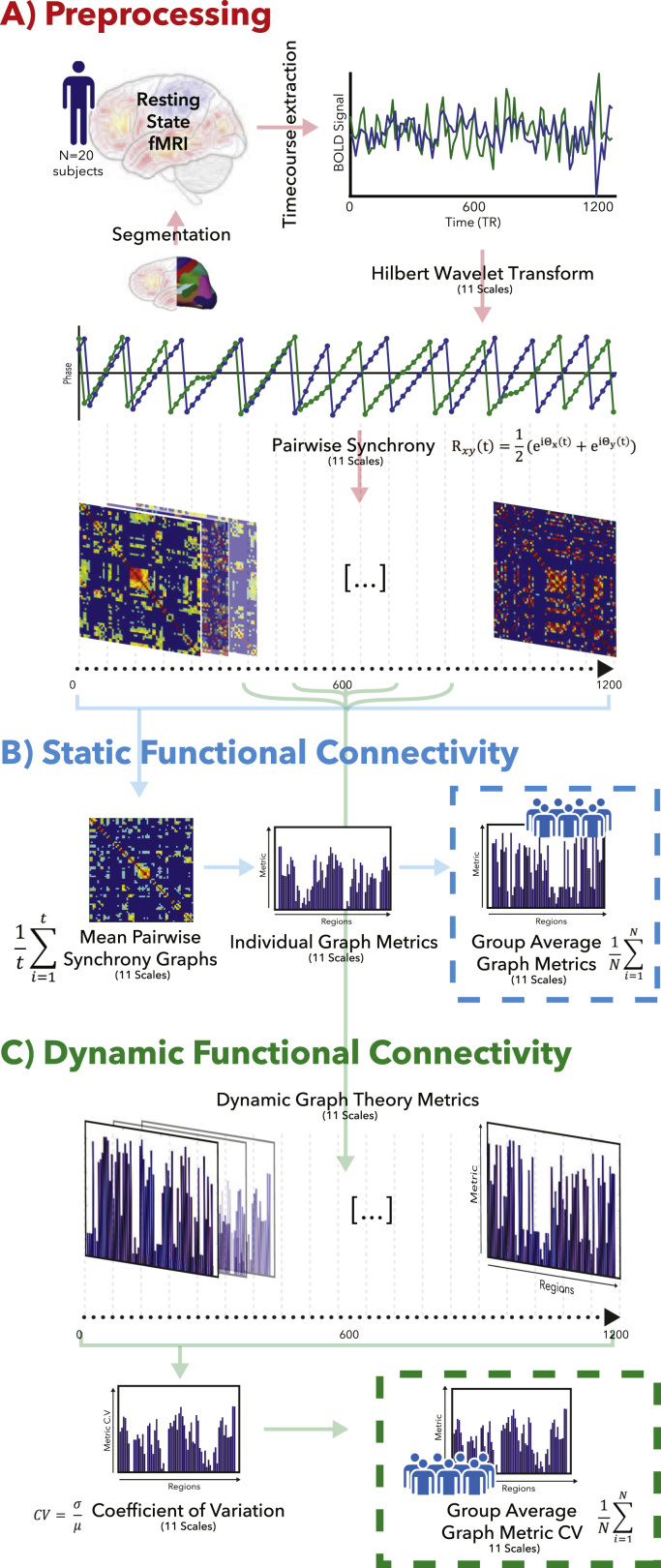
**Workflow describing the generation of the population network indexes.** R-fMRI data were pre-processed by extracting ROI data for each time-course, and transforming into 11 temporal ‘scales’ using the MODWT transform (See Materials and Methods). Two representative signals are displayed in the figure at a single scale (A). For each subject and at each wavelet scale we generated a time-dependent phase synchronisation matrix (C). Network Metrics were then calculated either for a temporal mean of the phase synchrony matrix (B), or for each time-point in the experimental data (C). For dynamic data stability of each metric were calculated across time (Coefficient of Variation – CV) for each subject and scale. For both static and dynamic measures, a population mean was generated across all N subjects for further analysis.

Following acquisition R-fMRI data were re-constructed and corrected for artefacts including motion according to the standard HCP FIX pipeline (Glasser et al., 2013, Griffanti et al., 2014, Salimi-Khorshidi et al., 2014, Smith et al., 2013, Van Essen et al., 2012), resulting in relatively artefact-free functional data co-registered into the MNI-152(2mm) coordinate system. R-fMRI data were segmented into the same 82 regions of interest (ROIs) described above for each PET atlas. For R-fMRI, we extracted time-courses for each region, relating the spatial average BOLD signal within each ROI at each time point, resulting in an 82(n)x1200(t) matrix for each R-fMRI dataset (Fig. 1A). To ensure consistency of the results across a range of different parcellation schemes, all analysis was repeated with two additional parcellation schemes (see Supplementary methods and results).

#### Functional connectivity

Measures of functional connectivity, were derived using a used a phase-synchronisation approach applied to the ROI data extracted from each resting state run (Ponce-Alvarez et al., 2015). In order to evaluate phase synchrony of the analytical signal derived from each of the ROI time courses across a broad-band of temporal scales, we derived the analytical signal across time in each ROI from the coefficients of a wavelet filter bank using wavelets derived as approximate Hilbert Wavelet Pairs (HWP) (Selesnick, 2001, Selesnick, 2002, Whitcher et al., 2005), as described in (Kitzbichler and Bullmore, 2015, Kitzbichler et al., 2009). HWP analysis was performed using a maximum overlap, discreet wavelet packet transform (MODWT) (Percival and Mofjeld, 1997, Percival and Walden, 2000), at 11 temporal scales (Fig. 1A). This resulted in phase representations within discreet dyadic ranges of the frequency spectrum of the underlying data: Scale (Hz); 1 (0.7–1.4), 2 (0.35–0.7), 3 (0.17–0.35), 4 (0.08–0.17), 5 (0.04–0.08), 6 (0.02–0.04), 7 (0.01–0.02), 8 (0.005–0.01), 9 (0.0025–0.005), 10 (0.0013–0.0025), 11 (0–0.0013). Strictly speaking, the frequency response at scale 1 is above the Nyquist frequency of our dataset, and higher scales e.g. 9-11, represent very low frequency signals given the short period of rest fMRI data considered. we include these scales for completeness, and are included and presented in supplement to the main results which in all figures are shown in heavy print. Code for performing the Hilbert-wavelet packet transform is available on GitHub: https://github.com/petehellyer/Phase_Sync_Analysis. To reduce computational complexity, and due to rapid noise fluctuations inherent in the estimation of the instantaneous analytical signal, a more tractable signal was derived, by decimating the phase estimates derived from the HWP filtering regime by a factor of 4 using an appropriate finite impulse response (FIR) filter. To explore time dependent measures of phase synchronisation, we employ a phase synchronisation methodology based on the widely used Kuramoto order parameter (Cabral et al., 2011, Cabral et al., 2014, Hellyer et al., 2015, Hellyer et al., 2014, Shanahan, 2010, Vasa et al., 2015, Wildie and Shanahan, 2012). For each pair of ROIs, a time dependent measure of phase synchrony between each analytical time course (Θ) was estimated using the pairwise form of the Kuramoto order parameter R(t) defined by:

(1) $$R_{\mathrm{xy}}\left(t\right)=\frac{1}{2}(e^{i\Theta_{x}\left(t\right)}+e^{i\Theta_{y}\left(t\right)})$$

where $\Theta_{x}$and $\Theta_{x}$ are the instantaneous phase of the analytical signal for one pair of ROIs. This results for each wavelet scale in a time-dependent matrix of pairwise synchrony between each of the ROIs considered. Static measures of FC (time averaged FC) were derived by taking the temporal average of $R_{\mathrm{xy}}\left(t\right)$ (Fig. 1B), dynamic measures of FC were defined as the square matrix R at each time point (dynamic FC – Fig. 1C).

#### Graph theoretical metrics

For both time-averaged FC and dynamic measures of FC, network-based graph theoretical metrics were calculated using the Brain Connectivity Toolbox. We included Clustering Coefficient, Local Efficiency, Node Strength and Betweenness Centrality:

• **Clustering Coefficient:** as a measure of functional segregation, it is based on the number of triangles in the network, with a high number of triangles implying segregation. Locally, the number of triangles around an individual node over the number of connected triples is known as the clustering coefficient.
• **Local Efficiency:** is the ratio of the number of connections between a node’s neighbours to the total number of possible links.
• **Node Strength:** is the average connectivity of a node. Strength is the weighted variant of the degree, and is defined as the sum of all neighbouring link weights.
• **Betweenness Centrality:** is an indicator of the centrality of a node within the network and is calculated as the number of shortest paths between all nodes that pass through a specific node.

It is to be noted however, that these four measures are not necessarily orthogonal – This effectively separates these four measures into two categories – one describing integration of nodes (Clustering Coefficient, Local Efficiency and Betweenness Centrality) and one describing overall connectivity of individual nodes (Node Strength). Whilst comparisons between measures are therefore not entirely independent, we present all analysis for completeness.

In order to select an appropriate threshold for graph theoretical analysis, we sought to select an appropriate threshold for each graph that maximised the Cost-Efficiency (*C-E*) of each resulting network (Achard and Bullmore, 2007). This threshold was calculated for each static graph (weighted) at each scale in an independent group of 20 controls drawn from the HCP. The mean threshold across each of these subjects was then applied in the static and dynamic analysis in the main study cohort; the thresholds used are reported in Supplementary Fig. S7. We next computed the measures above for each individual network, obtaining 20 sets of graph-theoretical measures for each atlas and each filtering band. As last step, we averaged the network measures across subjects, obtaining population averages of each graph theoretical index (Fig. 1A). For each time-point at each temporal scale, we calculated graph theoretical measures as described above. Variability in local node-metrics across the 15-min time interval was then evaluated using the coefficient of variation (CV) between (i.e. standard deviation/mean) of each metric for each node (Fig. 1C). Finally, these single subject CV were then averaged between subjects to generate an overall grand-mean variability of each metric across the brain. Graph theoretical measures estimated at each temporal scale of the Hilbert-wavelet pairs transform were associated with measures of [^18^F]FDG SUV and [1-^11^C]Leucine rCPS, using repeated measures general linear modelling. Significance of each factor within the repeated measures design was assessed using F statistics. Assumptions of Sphericity were tested using Mauchlys criteria and, where appropriate, significance and degrees of freedom of within-subject effects are reported for the conservative Greenhouse-Geisser correction.

## Results

### Metabolism measured using [^18^F]FDG SUV is enhanced in functional ‘hub’ nodes

Consistent with previous literature (Aiello et al., 2015, Riedl et al., 2014), we began by exploring the extent to which functional ‘hubs’ in the brain correlate with local measures of metabolism measured using [^18^F]FDG SUV. We started by calculating average measures of Node Strength, Clustering Coefficient, Local Efficiency and Betweenness Centrality in the cortex from the R-fMRI data in each of the 11 temporal scales considered. Repeated measures linear modelling demonstrated a significant relationship between [^18^F]FDG SUV and Clustering Coefficient (F_1,80_=41.36, p<0.001), Node Strength (F_1,80_=12.66, p<0.001),

Local efficiency (F_1,80_=32.48, p<0.001) and Betweenness Centrality (F_1,80_=5.41, p<0.05).

There was a significant interaction between temporal scale and Clustering Coefficient (F_2150_=23.48, p<0.001), Node Strength (F_3216_=2.51, p<0.01) ,Local Efficiency (F_2135_=21.34, p<0.001) and Betweenness Centrality (F_3250_=2.52, p<0.001).

**Fig. 2:**
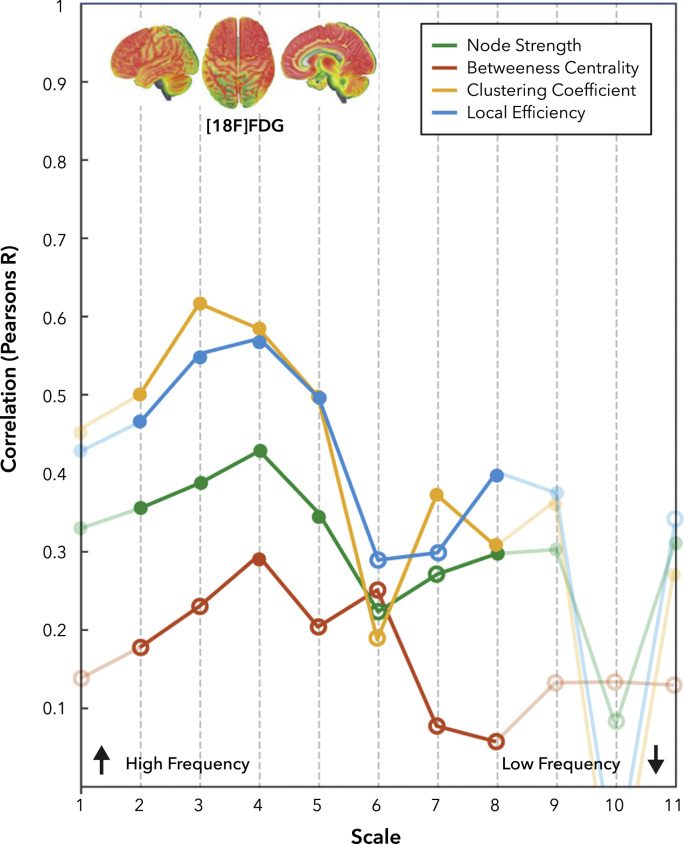
Correlation values between R-fMRI network measures and [^18^F]FDG SUV, as function of BOLD scale. Filled nodes are significant (p<0.05 – Bonferroni Corrected).

A high-level overview of these results is shown in Table 1A, however to explore these effects in more detail, we performed post-hoc correlations (as Pearson’s correlation coefficient) between network indices of centrality and [^18^F]FDG SUV (Fig. 2). Whilst there was a significant relationship between [^18^F]FDG SUV and graph theoretical measures across all frequency bands, there is also a clear modulation in terms of temporal scale. To assess the specificity of these results to the specific structural segmentation scheme, we repeated this analysis with two other unique segmentations (Supplementary Fig. S2). In addition to explore the effect of gender and sampling bias in the fMRI dataset, we repeated the analysis for an additional 20 subjects from the HCP cohort as well as two randomly selected male and female cohorts from the HCP (Supplementary results 1); with qualitatively similar results.

### Protein turnover measured using [1-11C]Leucine is correlated with functional ‘hub’ nodes

Next, we directly explored the relationship between protein synthesis in the cortex and local measures of network connectivity. Reconstructed statistical maps at each temporal scale for each of the graph theory metrics discussed are attached as interactive 3D models. Repeated measures linear modelling demonstrated a significant relationship between [1-^11^C]Leucine rCPS and Betweenness Centrality (F_1,80_=18.34, p<0.001), Node Strength (F_1,80_=13.68, p<0.01), Clustering Coefficient (F_1,80_=95.93, p<0.001) and Local efficiency (F_1,80_=91.70, p<0.001). There was a significant interaction between temporal scale and Betweenness Centrality (F_3,265_=6.19, p<0.001), Node Strength (F_3,227_=9.71, p<0.001), Clustering Coefficient (F_2,179_=52.38, p<0.001) and Local Efficiency (F_2,151_=48.98, p<0.001). These effects are explored in more detail using post-hoc correlation between network indices and [1-^11^C]Leucine rCPS in Supplementary Fig. 3.

One possible explanation for strong associations between both [1-^11^C]Leucine rCPS, and [^18^F]FDG SUV and graph theoretical measures may be that protein synthesis is directly associated with high metabolic rate or vice versa. Indeed, both binding measures are significantly correlated (r=0.63, p<0.001). We extended our analysis to specifically control for this putative relationship. In a combined model using [^18^F]FDG SUV and [1-^11^C]Leucine rCPS as predictors simultaneously, we observed main effects associations of [1-^11^C]Leucine rCPS with Betweenness Centrality (F_1,79_=12.04, p<0.001), Local Efficiency (F_1,79_=44.24, p<0.001), Clustering Coefficient (F_1,79_=41.79, p<0.001), There was no main effect of Node Strength (F_1,79_=3.54), though in the replication sets this main effect was present (Supplementary results 1).

In this model, there was no association between [^18^F]FDG SUV and Betweenness Centrality (F_1,79_=0.06), Local Efficiency (F_1,79_=1.74) and Node Strength (F_1,79_=3.54). There was a significant association between [^18^F]FDG SUV and Clustering Coefficient (F_1,79_=4.32, p<0.05).

Interactions between temporal scale and [^11^C]Leucine rCPS remained significant for.

Local Efficiency (F_2,146_=23.85, p<0.001), Clustering Coefficient (F_2,171_=25.34, p<0.001) and.

Betweenness Centrality (F_3,260_=3.97, p<0.001) and Node Strength (F_3,224_=10.39, p<0.001).

**Fig. 3:**
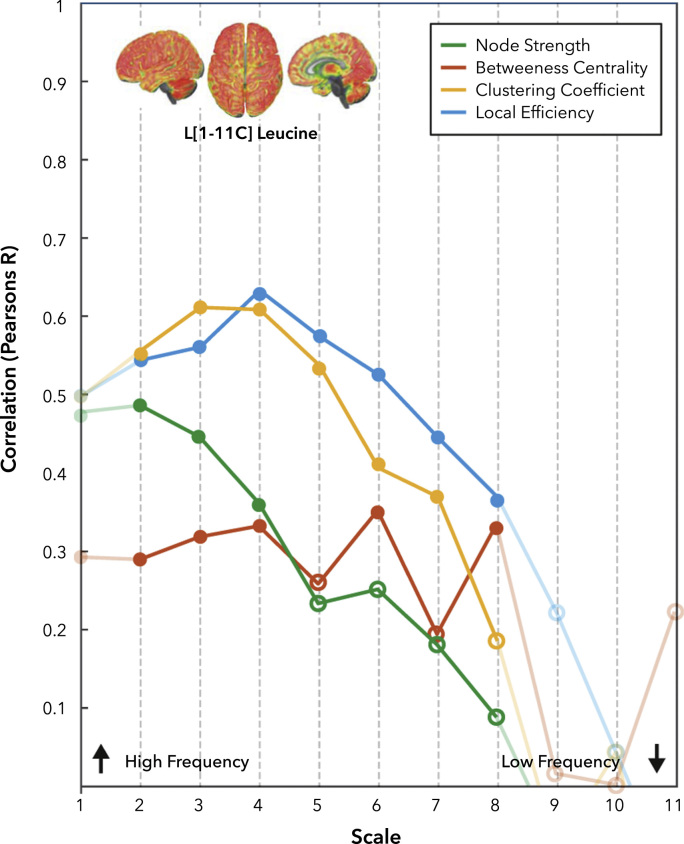
**Partial Correlation values between R-fMRI network measures and rCPS, as function of BOLD scale.** The correlation values are calculated as partial correlations, considering [^18^F]FDG SUV as a covariate of no interest. Filled nodes are significant (p<0.05 – Bonferroni Corrected).

A high-level overview of these results is shown in Table 1B, however to explore these effects in more detail, we performed post-hoc partial regressions between network indices of centrality and [1-^11^C]Leucine rCPS (Fig. 3). Like our associations with [^18^F]FDG SUV, whilst a significant association between [1-^11^C]Leucine rCPS and graph theoretical measures was observed all frequency bands, there is also a clear modulation in terms of temporal scale for Node Strength, Local Efficiency and Clustering Coefficient. These results were also qualitatively similar across a range of different segmentation schemes (Supplementary fig. 4).

### Stability of network hubs is inversely proportional to protein turnover measured using [1-11C]Leucine

**Fig. 4:**
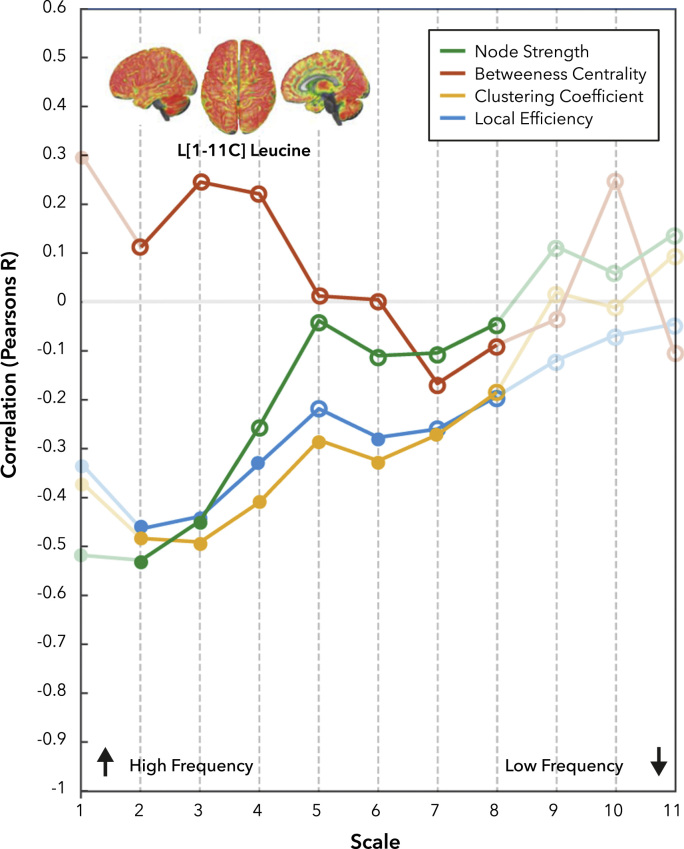
**Correlations between the CV of functional network measures and rCPS values as function of scale.** We compared the coefficient of variation over time of the network metrics obtained from R-fMRI versus rCPS measured with L-[1-^11^C]leucine considering [^18^F]FDG SUV as a covariate of no interest. Filled nodes are significant (p<0.05 – Bonferroni Corrected).

In the previous analysis, we considered only static measures of network connectivity over time. Functional connectivity however likely evolves dynamically over time (Calhoun et al., 2014, Monti et al., 2014, Sakoglu et al., 2010). Reconstructed statistical maps at each temporal scale for the CV over time of each graph theory metrics discussed are attached as interactive 3D models. To explore such a dynamic measure of connectivity over time, we adapted the phase synchronisation approach described above to measure time-varying phase synchronisation for each wavelet scale. At each time point in each scale, we estimated graph theoretical descriptions of functional connectivity (Fig. 1C). We used these estimates to explore how temporal stability of ‘hub’ measures, estimated using the temporal coefficient of variation (CV), relate to [1-^11^C]Leucine rCPS. We found that general highly connected regions of the brain show greater stability over time, particularly at higher temporal resolutions (Supplementary Fig. S6). This stability of network hub-ness was inversely related to [1-^11^C]Leucine rCPS (Fig. 4). High-level repeated measures linear modelling using [^18^F]FDG SUV and [1-^11^C]Leucine rCPS as predictors simultaneously demonstrated a significant relationship between [1-^11^C]Leucine rCPS and CV of Clustering Coefficient (F_1,79_=4.17 p<0.05) and Local efficiency (F_1,79_=7.02 p<0.01). There was no main effect relationship between Node Strength (F_1,79_=1.23) or Betweenness Centrality (F_1,79_=0.83). No main effect relationship in this combined model was observed between [^18^F]FDG SUV and CV of Betweenness Centrality (F_1,79_=1.34), Node Strength (F_1,79_=1.94), Clustering Coefficient (F_1,79_=2.61) and Local efficiency (F_1,79_=2.49). Finally, there was a significant interaction between temporal scale and [1-^11^C]Leucine rCPS for Betweenness Centrality (F_7,564_=2.64, p<0.05), Node Strength (F_4,284_=7.00, p<0.001), Clustering Coefficient (F_5,365_=2.52, p<0.01) but not Local Efficiency (F_4,338_=0.35). Similar, results were also seen across a range of other parcellation schemes (Supplementary Fig. 5). In addition to explore the effect of gender and sampling bias in the fMRI dataset, we repeated the analysis for a subset of male and female subjects, as well as an additional 20 subjects from the HCP cohort (Supplementary results 2); with qualitatively similar results (however see discussion).

## Discussion

The traditional view of the brain as a collection of functionally distinct regions operating in isolation has gradually given way in the past decade to a more holistic description of the brain as a highly connected network of functionally inter-connected modules which adapt in both the temporal and spatial domain in response to changes to both the external and internal environment (Corbetta and Shulman, 2002, Fornito et al., 2012, Fox et al., 2005, Hellyer et al., 2015, Vincent et al., 2008). Plasticity within the central nervous system (CNS) is likely mediated by myriad different mechanisms which are the consequence of both structural and functional reorganisations at the synaptic level. Indeed, a wealth of work demonstrates the importance of protein synthesis and reorganisation of local networks because of learning and memory (Flexner et al., 1962, Flexner et al., 1965, Flexner et al., 1964, Hernandez and Abel, 2008, Jarome and Helmstetter, 2014, Lopez et al., 2015, Nader et al., 2000). The level to which plasticity within the CNS relates to macroscopic level neural dynamics at faster timescales is however unclear. Previous theoretical and experimental work suggests that local plasticity is an important mechanism by which rich spontaneous dynamics are organised within the brain (Cowan et al., 2013b, Fornito et al., 2012, Hellyer et al., 2016, Magnasco et al., 2009). This suggests that rather than acting purely as a mechanism for long-term reorganisation and consolidation of learnt information, plasticity must effect change across a range of different spatial and temporal scales, from very fast to very slow dynamics.

The brain uses myelination to set boundaries to network plasticity; these boundaries appear to be looser for local networks, that are wired through high frequency activity, and less so for long-distance hubs that are characterized by transmission on low-frequency band. This phenomenon has been described as “meta-plasticity”, studied in pre-clinical and computational models but never demonstrated in human brain networks (Abraham, 1999, Abraham and Bear, 1996, Abraham and Tate, 1997, Zenke et al., 2013). From this association, we may assume that long-distance connections that broadly operate at a low temporal frequency (Beckmann et al., 2005, De Luca et al., 2006) are likely constrained largely by myelination. Plasticity at this more local level is therefore likely to be associated with higher-speed functional dynamics. Hence, we have also hypothesized that rCPS would show association to graph theoretical measures that would vary with the frequency range of fMRI oscillations, with the association being stronger at higher frequency bands. In this work, we explicitly attempted to explore the interaction between neural plasticity within the CNS (measured using an estimate of protein synthesis – L-[1^11^C]-Leucine PET) and macroscopic measures of network integration across a broad-range of temporal scales. We explored the local clustering coefficient – a measure which describes the affinity for any individual region of the brain to embed within tight-knit clusters and strength – where the overall connectivity is the sum of each links with the rest of the network. Regions with low clustering and high strength are therefore likely to be the ‘hub nodes’ of the brain, central to the efficient and effective distribution of information across the brain. We furthermore demonstrate a tendency for these relationships to be somewhat enhanced at higher temporal scales associated with functional dynamics in BOLD data (on the temporal order of 10–20 s, as opposed to minutes). We go on to demonstrate that the stability of graph theoretical metrics within each region of the brain is inversely proportional to protein-turnover – suggesting that whilst strongly interconnected regions at fast timescales are strongly associated with enhanced protein synthesis. Our data suggest that these highly-connected regions of the brain show enhanced stability over time. That highly connected regions are more stable, may be unsurprising given the statistical bounds inherent to functional connectivity measures (Betzel et al 2016; Thompson & Franson et al 2015). It may be that the inverse relationship that we demonstrate between rCPS and topological stability is influenced by this. However, whilst some degree of statistical interdependence cannot be ruled out, examination of the post-hoc modelling suggests that rather than being a simple inverse of the static findings a different pattern of negative correlations emerges between stability and rCPS to the static analysis, supporting a hypothesis that those regions whose interconnectivity is relatively stable are associated with less protein turnover overall.

To some extent we replicate the previous findings in the literature that demonstrated that hub-regions of the brain are associated with high metabolic demand – specifically, measures of local integration and degree are strongly associated with [^18^F]FDG PET binding (Passow et al., 2015, Riedl et al., 2014, Tomasi et al., 2013). This suggests that informational hubs within the brain are highly metabolic, and somewhat mirrors the relationship that we describe rCPS. The causality between these two observations is unclear. To explore the relationship between protein turnover and metabolic rate we calculated the Pearson’s correlation coefficient between rCPS and SUV measures in the same regions of interest. We found that high levels of protein synthesis are associated with high metabolism.

Free leucine has been suggested as a major donor of amino acids for the synthesis of glutamate within the brain (Yudkoff et al 2005) – as increased metabolic load would be associated with glutamate production, it follows that increased metabolic load in local regions would similarly be associated with measures of protein turnover – accounting for a similarity in the relationship between rCPS and SUV across a range of topological and connectivity measures. However, our results suggest not that a strong association between rCPS and network connectivity is clearly present even when the effect of [^18^F]FDG binding is accounted for, but also that rCPS variability accounts for most of the association between [^18^F]FDG and brain dynamics is largely accounted for by the effect of rCPS. Hence rCPS is elevated in hub regions beyond that what might be expected simply because of ‘enhanced’ metabolism within these regions.

In many graph, theoretical explorations of the brain, one significant factor in the associations generated is the specific parcellation scheme used. To counter this problem, we repeated the clear majority of our post-hoc correlations for two alternative parcellation schemes (Craddock et al., 2012; Tzourio-Mazoyer et al., 2002) with qualitative similarities across all three schemes (Supplementary figs. 2,4,5). The segmentation employed to define the regions of interest (and therefore the nodes of the network) however did have a subtle impact on the final correlation values. The AAL atlas showed marginally stronger correlations (albeit still significant for most of the lower frequencies) compared to Craddock and Freesurfer atlases. Since the metabolism in the brain is most intense in the cortical areas, the looser cortical parcellation of the AAL atlas may explain differences in correlations between graph metrics and SUV values. Secondly, the choice of metric to describe functional connectivity may have an impact on the overall graph theoretical analysis. In this work, we chose to use a phase synchronisation approach. This was made technically feasible by the enhanced temporal resolution of the multi-band resting state acquisition in the Human Connectome Project dataset. However, approaches such as pair-wise correlation or partial correlation using the general linear model may permit alternative interpretations of the graph theoretical analysis which may affect the relationships described here.

The association that we describe here is inherently cross-sectional. This is in part due to an attempt to combine information from a broad-range of multi-modal and unique datasets – indeed, the L-[1^11^C]-Leucine data that we use here is to our knowledge a unique dataset which is not available elsewhere. Due to limitations in the data available, we were not able to fully match the groups in the analysis for both age and gender – for example, the L-[1^11^C]-Leucine data were collected only in male subjects, whilst the [^18^F]FDG PET data were acquired in a slightly older group of subjects. Whilst the collection of within subject data would improve the efficiency of our results, when we were able to re-analyse the existing dataset – in the case of age and gender matched groups for [^18^F]FDG PET, results remained stable. Equally, when female participants were removed from the [1-^11^C]Leucine PET analysis (and replaced by additional male subjects drawn from the HCP, results remained stable) (Supplementary results 1/2). An ideal situation would be to obtain functional, structural and PET data within the same subjects, and perform a true within-subject comparison between dynamics and measures of metabolism and protein synthesis. This is the focus of ongoing work and would be an ideal application for combined PET/MRI acquisition. Nevertheless, this work provides a proof-of principle of a putative relationship between protein synthesis, metabolism and broad-band neural dynamics.

## Disclosure

The authors declare no relevant financial interests or conflicts of interest.

## Author contributions

PJH, MV, GR & FET designed the research. PJH, AP, EB, MV, GR, MT AB & FET performed the research. MS, MB and MAR Provided un-published data and analysis methods. All authors wrote and/or edited the paper.

## Footnotes

Appendix A Supplementary data associated with this article can be found in the online version at doi:10.1016/j.neuroimage.2017.04.062.
