# Supplementary material for: Protein synthesis is associated with high-speed dynamics and broad-band stability of functional hubs in the brain
Source: Neuroimage. 2017 Jul 15;155:209–16. doi: 10.1016/j.neuroimage.2017.04.062 (PMC5519503; doi:10.1016/j.neuroimage.2017.04.062)
Supplement: Figure S1 — Supplementary material [file mmc1.pdf]

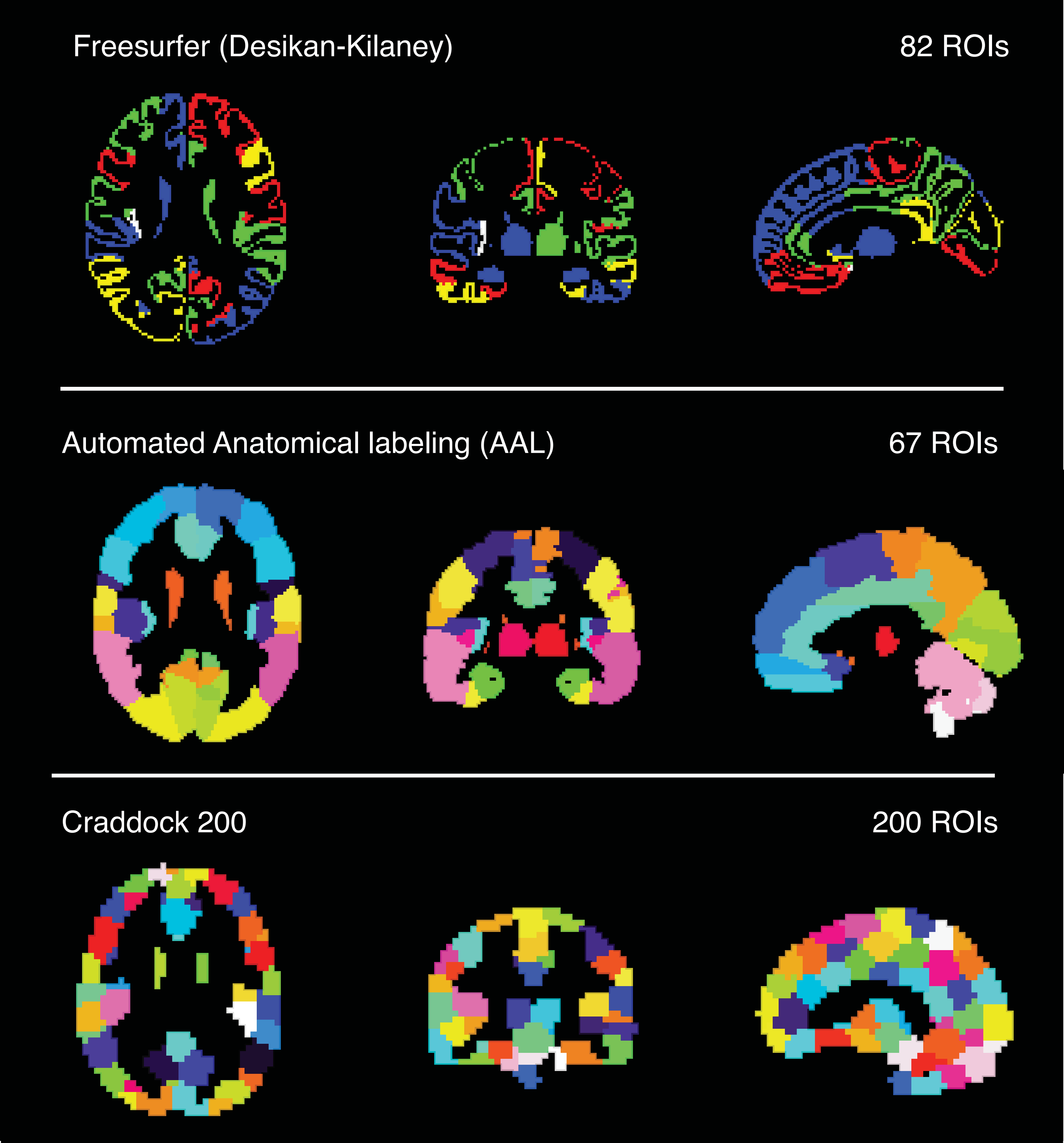
Freesurfer (Desikan-Kilaney)
82 ROIs
Automated Anatomical labeling (AAL)
67 ROIs
Craddock 200
200 ROIs

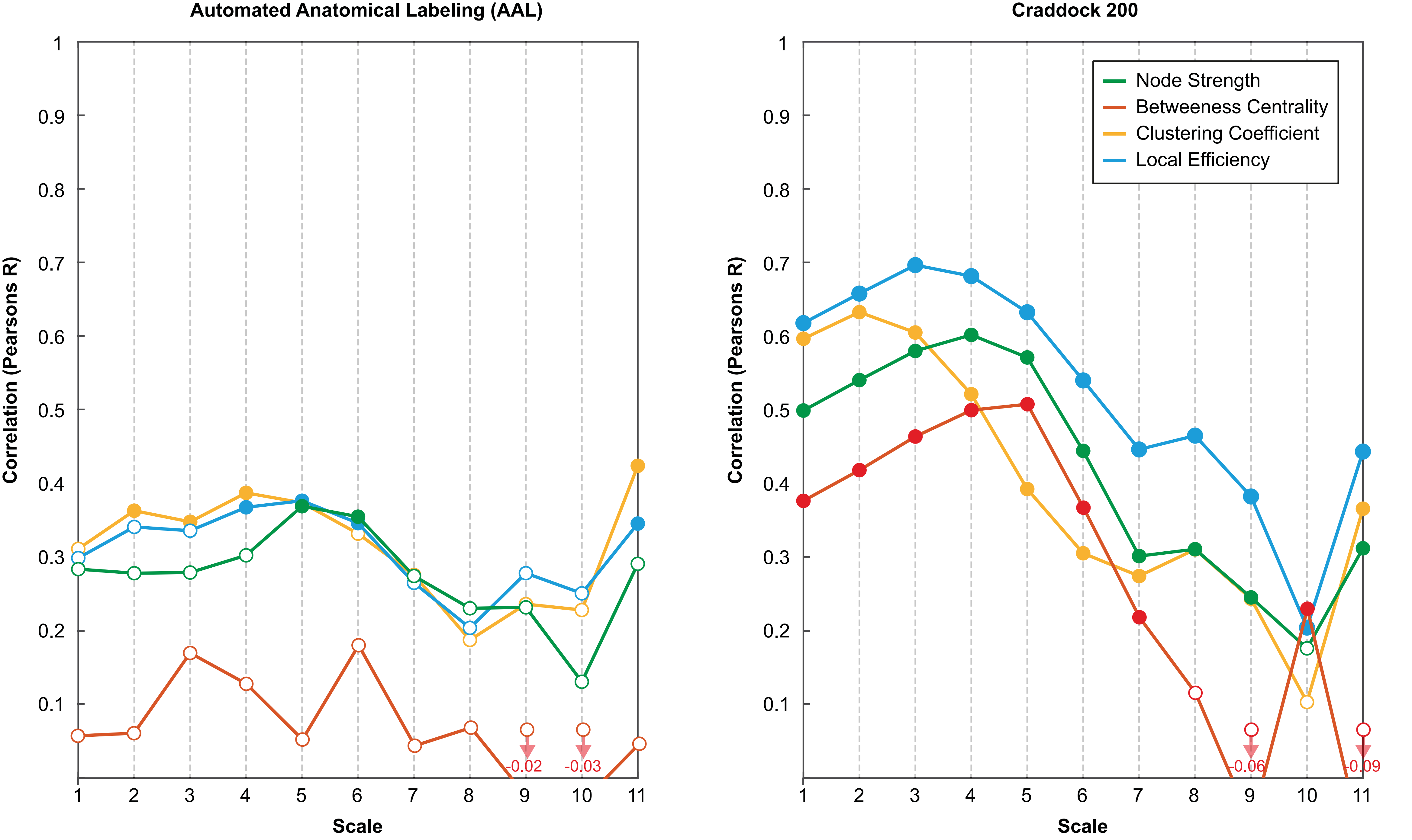

Automated Anatomical Labeling (AAL)
Craddock 200
Correlation (Pearsons R)
Scale
Node Strength
Betweeness Centrality
Clustering Coefficient
Local Efficiency
-0.02
-0.03
-0.06
-0.09

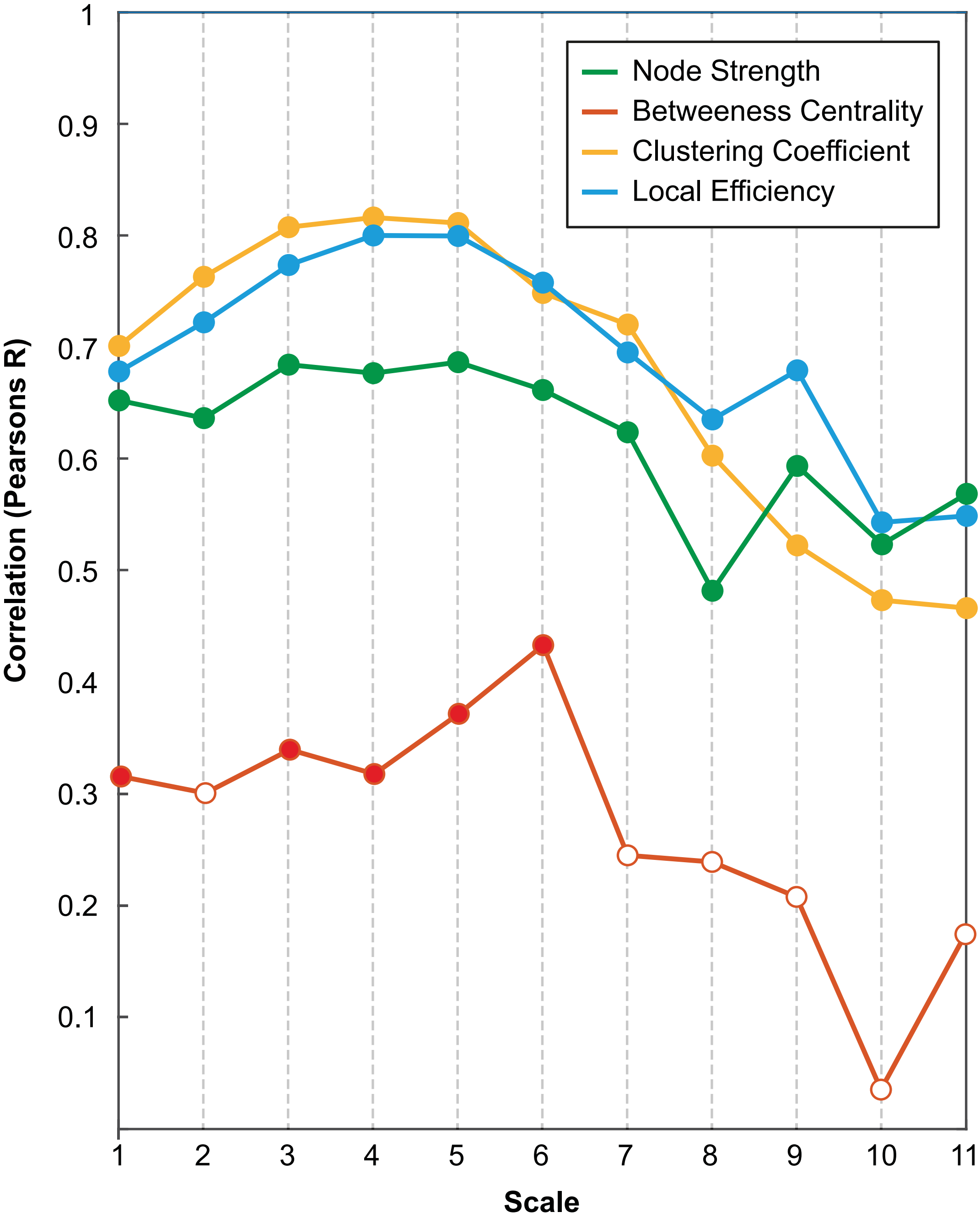

Node Strength
Betweeness Centrality
Clustering Coefficient
Local Efficiency
Correlation (Pearsons R)
1
0.9
0.8
0.7
0.6
0.5
0.4
0.3
0.2
0.1
Scale
1
2
3
4
5
6
7
8
9
10
11

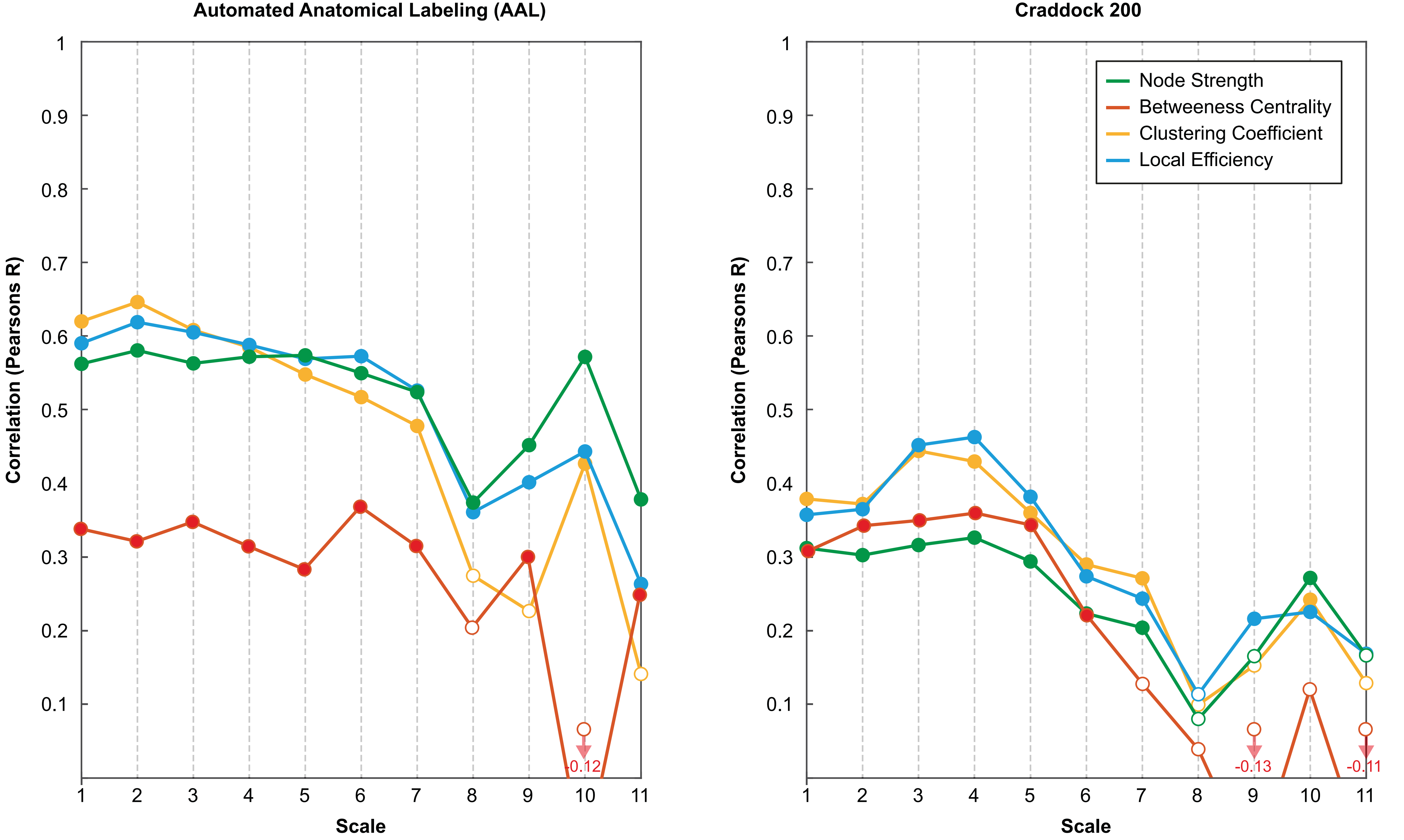

Automated Anatomical Labeling (AAL)
Craddock 200
Correlation (Pearsons R)
Scale
Node Strength
Betweeness Centrality
Clustering Coefficient
Local Efficiency
-0.12
-0.13
-0.11

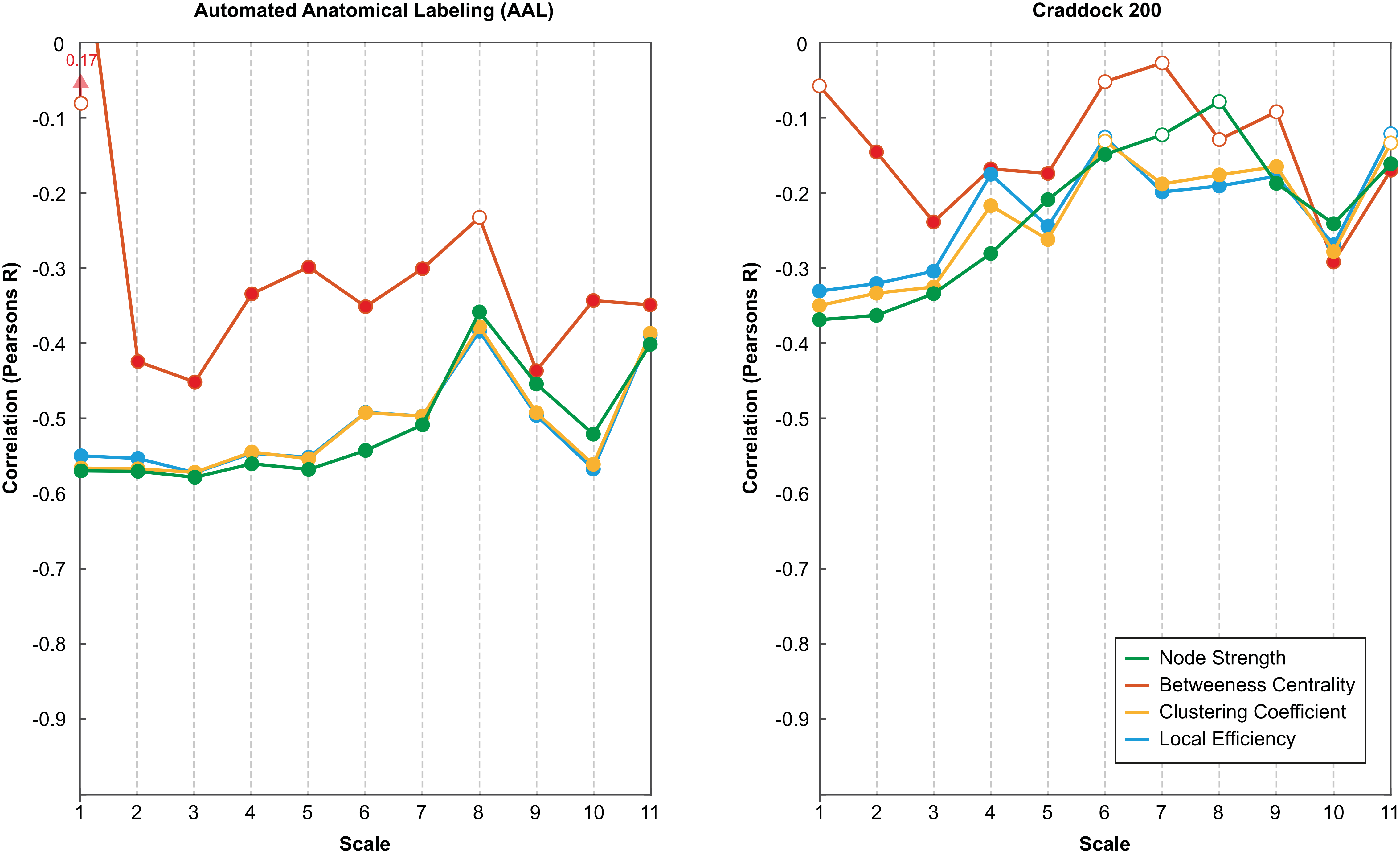
Automated Anatomical Labeling (AAL)
Craddock 200
Correlation (Pearsons R)
Scale
0.17
0
-0.1
-0.2
-0.3
-0.4
-0.5
-0.6
-0.7
-0.8
-0.9
1
2
3
4
5
6
7
8
9
10
11
Node Strength
Betweeness Centrality
Clustering Coefficient
Local Efficiency

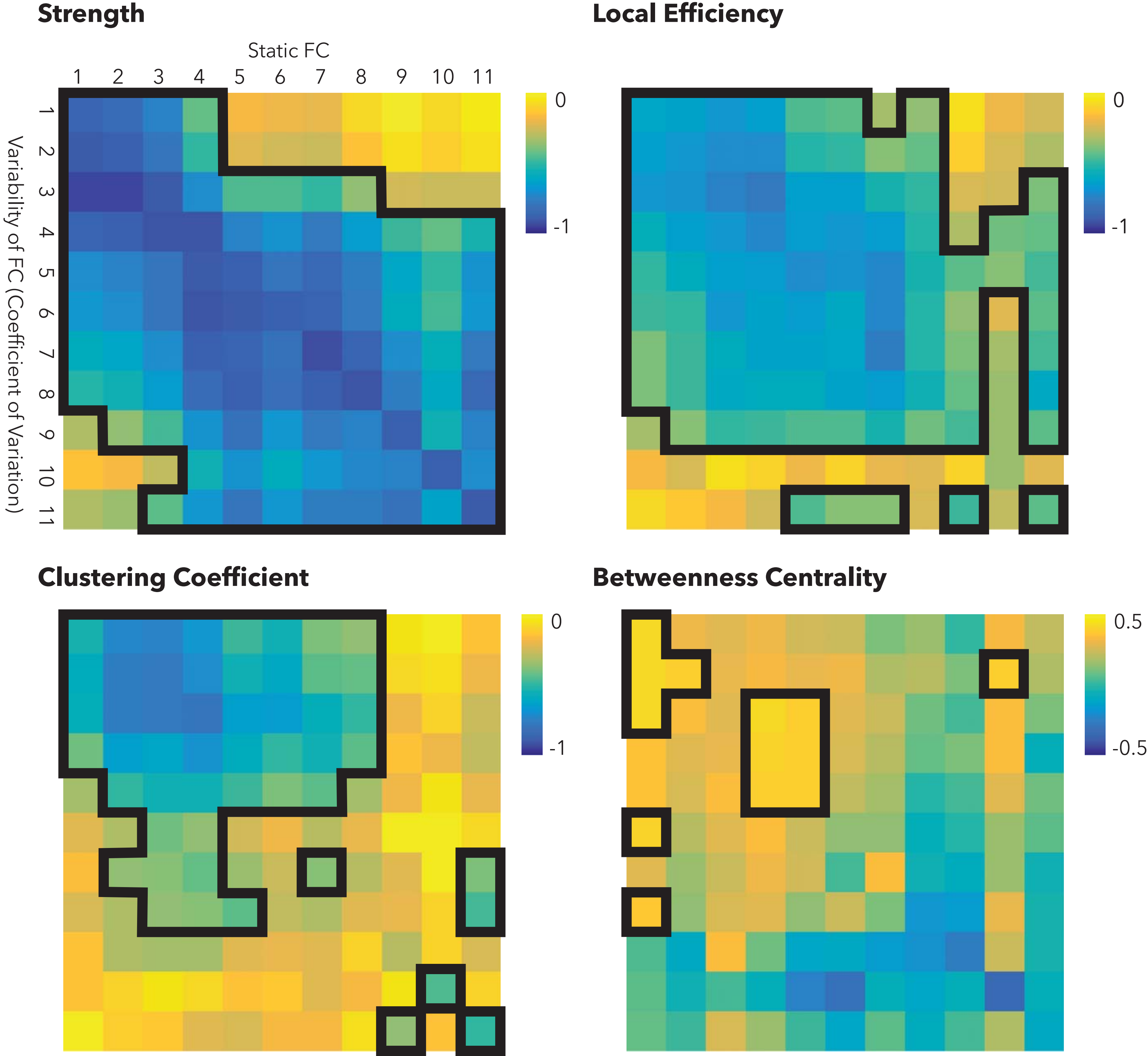
Strength
Static FC
1 2 3 4 5 6 7 8 9 10 11
Variability of FC (Coefficient of Variation)
1 2 3 4 5 6 7 8 9 10 11
0
-1
Local Efficiency
0
-1
Clustering Coefficient
0
-1
Betweenness Centrality
0.5
-0.5

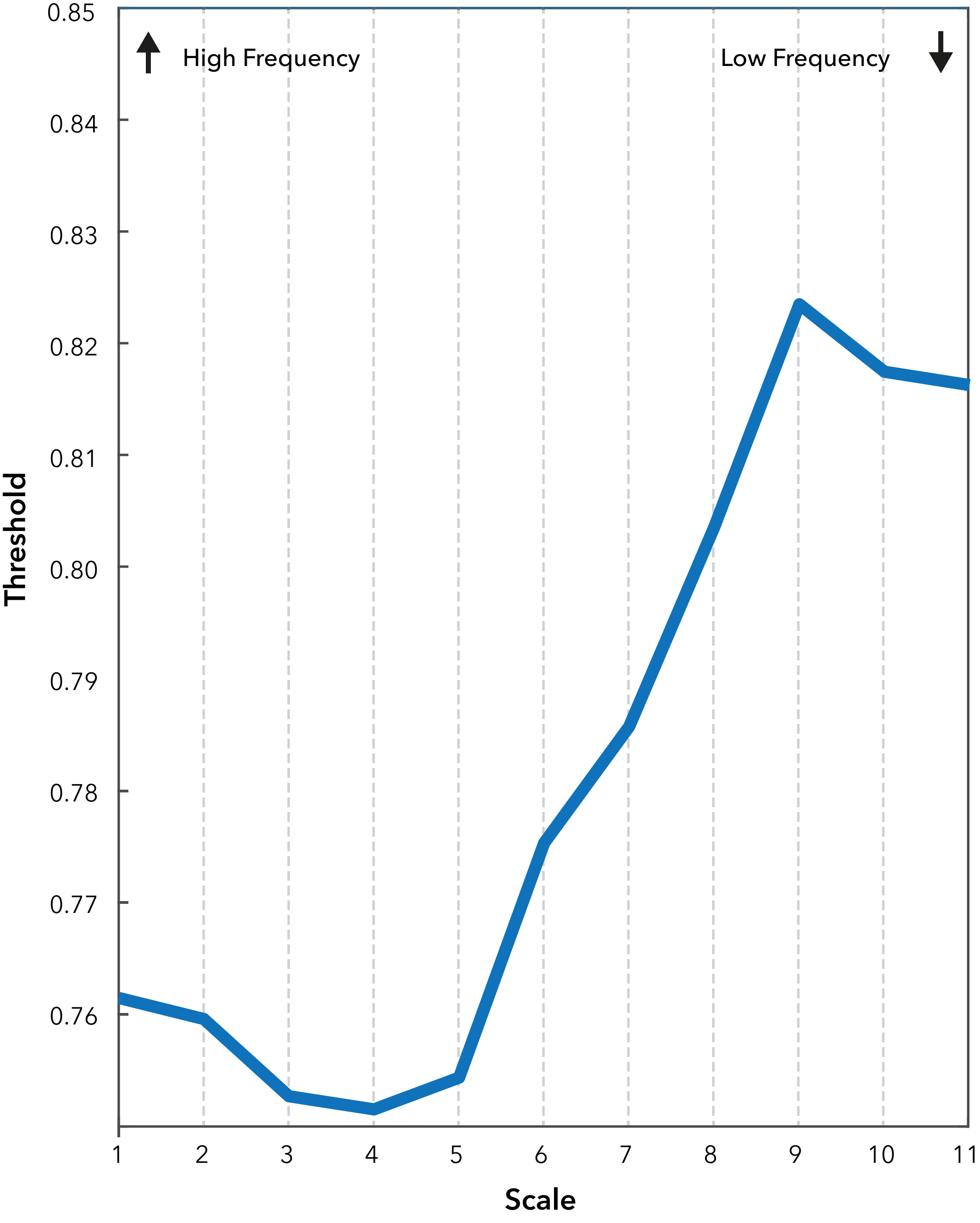
High Frequency
Low Frequency
Threshold
0.85
0.84
0.83
0.82
0.81
0.80
0.79
0.78
0.77
0.76
Scale
1
2
3
4
5
6
7
8
9
10
11
