# Supplementary material for: Protein synthesis is associated with high-speed dynamics and broad-band stability of functional hubs in the brain
Source: Neuroimage. 2017 Jul 15;155:209–16. doi: 10.1016/j.neuroimage.2017.04.062 (PMC5519503; doi:10.1016/j.neuroimage.2017.04.062)
Supplement: Figure S2 — Supplementary material [file mmc2.docx]

**Clustering Coefficient**

|  | rCPS | | [^18^F]FDG | | rCPS ( -[^18^F]FDG) | |
| --- | --- | --- | --- | --- | --- | --- |
|  | Interaction | Main Effect | Interaction | Main Effect | Interaction | Main Effect * |
| Group 1 | F_2,179_=52.38 | F_1,80_=95.93 | F_2,150_=23.48 | F_1,80_=41.36 | F_2,171_=25.34 | F_1,79_=41.79 |
| Group 2 | F_2,173_=56.56 | F_1,80_=106.55 | F_2,150_=26.82 | F_1,80_=40.98 | F_2,171_=25.09 | F_1,79_=48.42 |
| Male | F_2,178_=60.16 | F_1,80_=110.3 | F_2,150_=25.32 | F_1,80_=49.2 | F_2,173_=28.77 | F_1,79_=46.83 |
| Female | F_2,180_=48.51 | F_1,80_=97.59 | F_2,161_=27 | F_1,80_=44.24 | F_2,179_=19.72 | F_1,79_=41.46 |

* presented for rCPS only, main effects with [^18^F]FDG across groups (Group 1: F_1,79_=4.32 p<0.05; Group 2: F_1,79_=3.63 n.s.; Male: F_1,79_=6.44 p<0.05; Female: F_1,79_=5.27, p<0.05). p<0.001, p<0.01 p<0.05

**Node Strength**

|  | rCPS | | [^18^F]FDG | | rCPS ( -[^18^F]FDG) | |
| --- | --- | --- | --- | --- | --- | --- |
|  | Interaction | Main Effect | Interaction | Main Effect | Interaction | Main Effect * |
| Group 1 | F_3,227_=9.71 | F_1,80_=13.68 | F_3,216_=2.51 | F_1,80_=12.66 | F_3,224_=10.39 | F_1,79_=3.54 |
| Group 2 | F_3,277_=7.63 | F_1,80_=20.09 | F_3,278_=5.6 | F_1,80_=8.59 | F_3,268_=3.6 | F_1,79_=10.42 |
| Male | F_3,231_=10.07 | F_1,80_=15.3 | F_3,230_=5.85 | F_1,80_=11.04 | F_3,227_=5.82 | F_1,79_=5.23 |
| Female | F_4,290_=10.46 | F_1,80_=16.36 | F_4,281_=3.38 | F_1,80_=9.62 | F_4,284_=8.22 | F_1,79_=6.74 |

* presented for rCPS only, main effects with [^18^F]FDG n.s. across groups (Group 1: F_1,79_=2.65); Group 2: F_1,79_=0.14; Male: F_1,79_=1.47; Female: F_1,79_=0.75). p<0.001, p<0.01 p<0.05

**Local efficiency**

|  | rCPS | | [^18^F]FDG | | rCPS ( -[^18^F]FDG) | |
| --- | --- | --- | --- | --- | --- | --- |
|  | Interaction | Main Effect | Interaction | Main Effect | Interaction | Main Effect * |
| Group 1 | F_2,151_=48.98 | F_1,80_=91.7 | F_2,135_=21.34 | F_1,80_=32.48 | F_2,146_=23.85 | F_1,79_=44.24 |
| Group 2 | F_2,150_=53.14 | F_1,80_=100.28 | F_2,137_=24.68 | F_1,80_=36.23 | F_2,147_=23.95 | F_1,79_=47.29 |
| Male | F_2,150_=53.31 | F_1,80_=109.12 | F_2,136_=23.45 | F_1,80_=43.87 | F_2,145_=25.15 | F_1,79_=48.49 |
| Female | F_2,158_=48.22 | F_1,80_=87.63 | F_2,146_=24.21 | F_1,80_=34.57 | F_2,156_=21.15 | F_1,79_=40.29 |

* presented for rCPS only, main effects with [^18^F]FDG n.s. across groups (Group 1: F_1,79_=1.74; Group 2: F_1,79_=2.42; Male: F_1,79_=4.5 p< 0.05; Female: F_1,79_=2.53). p<0.001, p<0.01 p<0.05.

**Betweenness Centrality.**

|  | rCPS | | [^18^F]FDG | | rCPS ( -[^18^F]FDG) | |
| --- | --- | --- | --- | --- | --- | --- |
|  | Interaction | Main Effect | Interaction | Main Effect | Interaction | Main Effect * |
| Group 1 | F_3,265_=6.19 | F_1,80_=18.34 | F_3,250_=2.52 | F_1,80_=5.41 | F_3,260_=3.97 | F_1,79_=12.04 |
| Group 2 | F_3,246_=6.09 | F_1,80_=17.69 | F_3,235_=2.09 | F_1,80_=5.55 | F_3,243_=4.27 | F_1,79_=11.24 |
| Male | F_4,280_=5.6 | F_1,80_=16.31 | F_3,270_=3.22 | F_1,80_=6.66 | F_3,276_=2.72 | F_1,79_=8.85 |
| Female | F_4,287_=7.05 | F_1,80_=14.77 | F_3,263_=1.18 | F_1,80_=4.4 | F_4,284_=6.6 | F_1,79_=9.78 |

* presented for rCPS only, main effects with [^18^F]FDG n.s. across groups (Group 1: F_1,79_=0.06; Group 2: F_1,79_=0.03; Male: F_1,79_=0.05; Female: F_1,79_=0.34). p<0.001, p<0.01 p<0.05

**Supplementary Results 1:** Replication of the results reported in the main text for static functional connectivity data. Analysis was repeated for 4 Separate groups (n=20) derived from the Human Connectome Project (See Materials and Methods).

**Clustering Coefficient**

|  | rCPS ( -[^18^F]FDG) | | |
| --- | --- | --- | --- |
|  | Interaction | Main Effect ([^18^F]FDG) | Main Effect ([^18^F]FDG) |
| Group 1 | F_5,365_=2.52 | F_1,79_=5.17 | F_1,79_=2.61 |
| Group 2 | F_4,279_=1.20 | F_1,79_=9.8 | F_1,79_=0.67 |
| Male | F_5,387_=2.60 | F_1,79_=9.16 | F_1,79_=1.19 |
| Female | F_4,297_=2.19 | F_1,79_=6.44 | F_1,79_=1.1 |

p<0.001, p<0.01 p<0.05

**Node Strength**

|  | rCPS ( -^18^F]FDG) | | |
| --- | --- | --- | --- |
|  | Interaction | Main Effect (rCPS) | Main Effect ([^18^F]FDG) |
| Group 1 | F_4,284_=7.00 | F_1,79_=1.23 | F_1,79_=1.94 |
| Group 2 | F_4,288_=3.37 | F_1,79_=6.06 | F_1,79_=0.17 |
| Male | F_3,267_=4.29 | F_1,79_=2.87 | F_1,79_=1.61 |
| Female | F_4,280_=4.90 | F_1,79_=3.63 | F_1,79_=0.39 |

p<0.001, p<0.01 p<0.05

**Local efficiency**

|  | rCPS ( -[^18^F]FDG) | | |
| --- | --- | --- | --- |
|  | Interaction | Main Effect (rCPS) | Main Effect ([^18^F]FDG) |
| Group 1 | F_4,338_=0.35 | F_1,79_=7.02 | F_1,79_=2.49 |
| Group 2 | F_4,309_=2.57 | F_1,79_=15.58 | F_1,79_=0.05 |
| Male | F_4,354_=1.36 | F_1,79_=8.35 | F_1,79_=1.72 |
| Female | F_4,340_=3.62 | F_1,79_=10.58 | F_1,79_=0.78 |

p<0.001, p<0.01 p<0.05.

**Betweenness Centrality.**

|  | rCPS ( -[^18^F]FDG) | | |
| --- | --- | --- | --- |
|  | Interaction | Main Effect (rCPS) | Main Effect ([^18^F]FDG) |
| Group 1 | F_7,564_=2.64 | F_1,79_=0.83 | F_1,79_=1.34 |
| Group 2 | F_7,527_=1.75 | F_1,79_=1.41 | F_1,79_=1.78 |
| Male | F_7,566_=0.54 | F_1,79_=1.37 | F_1,79_=0.67 |
| Female | F_3,270_=2.79 | F_1,79_=5.8 | F_1,79_=1.49 |

p<0.001, p<0.01 p<0.05

**Supplementary Results 2:** Replication of the results reported in the main text for dynamic analysis of hub stability. Analysis was repeated for 4 Separate groups (n=20) derived from the Human Connectome Project (See Materials and Methods).
