# Supplementary material for: Protein synthesis is associated with high-speed dynamics and broad-band stability of functional hubs in the brain
Source: Neuroimage. 2017 Jul 15;155:209–16. doi: 10.1016/j.neuroimage.2017.04.062 (PMC5519503; doi:10.1016/j.neuroimage.2017.04.062)
Supplement: Figure S3 — Supplementary material [file mmc3.docx]

# Supplementary materials

**Figure S1. Anatomical atlases.** Three different anatomical atlases were included in the study: Automated Anatomical Labelling (AAL, top row), with 67 ROIs; Craddock atlas (middle row), with 200 ROIs; Freesurfer atlas (bottom row) with 82 ROIs.

**Figure S2. Correlations between functional network measures and [^18^F]FDG SUV.** Results are reported for two additional different parcellation schemes (Automated Anatomical Labelling (AAL) (Tzourio-Mazoyer N *et al.*) and Craddock 200 (Craddock RC *et al.*). Filled nodes are significant (p<0.05 – Bonferroni Corrected).

**Figure S3. Correlations between functional network measures and rCPS.** Results reported are raw correlation coefficients before correction for local metabolism using [^18^F]FDG SUV. Filled nodes are significant (p<0.05 – Bonferroni Corrected).

**Figure S4. Partial Correlation values between R-fMRI network measures and rCPS, as function of BOLD scale.** The correlation values are calculated as partial correlations, considering [^18^F]FDG SUV as a covariate of no interest. Results are reported for two additional different parcellation schemes (Automated Anatomical Labelling (AAL) (Tzourio-Mazoyer N *et al.* 2002) and Craddock 200 (Craddock RC *et al.*). Filled nodes are significant (p<0.05 – Bonferroni Corrected).

**Figure S5.** **Partial Correlation values between dynamic R-fMRI CV network measures and rCPS, as function of BOLD scale.** The correlation values are calculated as partial correlations, considering [^18^F]FDG SUV as a covariate of no interest. Results are reported for two additional different parcellation schemes (Automated Anatomical Labelling (AAL) (Tzourio-Mazoyer N *et al.*) and Craddock 200 (Craddock RC *et al.*). Filled nodes are significant (p<0.05 – Bonferroni Corrected).

**Figure S6. Correlation between static connectivity measures and variability in connectivity over time**. Graph theoretic metrics were re-constructed into spatial maps (See supplementary 3D figures) for both static graph theoretic metrics, and stability measures (Coefficient of variation across time). Here, correlation coefficients were calculated between stability and static graph theoretical measures in Group 1 (n=20). Statistically significant correlations (p<0.05 – Bonferroni corrected) are highlighted in bold.

**Figure S7. Thresholds for graph theoretical analysis** across 11 time-scales. Thresholds were calculated by maximising Cost-Efficiency (*C-E*) of each cross connectivity network (Achard and Bullmore, 2007) at each time-scale derived from 20 independent control subjects of the HCP dataset (See Materials and Methods).

Tables

1. [^18^F]FDG SUV

|  | Main Effect | Interaction |
| --- | --- | --- |
|  | **[^18^F]FDG SUV** | **[^18^F]FDG SUV * Scale** |
| Node Strength | **✓** | **✓** |
| Betweenness Centrality | **✓** | **✓** |
| Clustering Coefficient | **✓** | **✓** |
| Local Efficiency | **✓** | **✓** |

1. [1-^11^C]Leucine rCPS (Corrected for [^18^F]FDG SUV)

|  | Main Effect | | Interaction | |
| --- | --- | --- | --- | --- |
|  | **[1-^11^C]Leucine rCPS** | **[^18^F]FDG SUV** | **[1-^11^C]Leucine rCPS * Scale** | **[^18^F]FDG SUV * Scale** |
| Node Strength | ✕ | ✕ | **✓** | **✓** |
| Betweenness Centrality | **✓** | ✕ | **✓** | ✕ |
| Clustering Coefficient | **✓** | **✓** | **✓** | **✓** |
| Local Efficiency | **✓** | ✕ | **✓** | ✕ |

**Table 1: Overview of relationships between A) [^18^F]FDG SUV, B) [1-^11^C]Leucine rCPS and functional connectivity.** *Main Effect* and interaction of the main effect with temporal scale (*Interaction*) between functional connectivity across 11 different wavelet scales in Group 1. **✓**represents a statistically significant effect (p<0.05 – Bonferroni corrected), whereas ✕represents no significant effect. Where appropriate statistics from repeated measures ANOVA are reported in the main text.
